# Global Regulation of Nucleotide Biosynthetic Genes by c-Myc

**DOI:** 10.1371/journal.pone.0002722

**Published:** 2008-07-16

**Authors:** Yen-Chun Liu, Feng Li, Jesse Handler, Cheng Ran Lisa Huang, Yan Xiang, Nicola Neretti, John M. Sedivy, Karen I. Zeller, Chi V. Dang

**Affiliations:** 1 Program in Pathobiology, Johns Hopkins University School of Medicine, Baltimore, Maryland, United States of America; 2 Division of Hematology, Department of Medicine, Johns Hopkins University School of Medicine, Baltimore, Maryland, United States of America; 3 Program in Human Genetics and Molecular Biology, Johns Hopkins University School of Medicine, Baltimore, Maryland, United States of America; 4 The Sidney Kimmel Cancer Center at Johns Hopkins, Johns Hopkins University School of Medicine, Baltimore, Maryland, United States of America; 5 Department of Molecular Biology, Cell Biology and Biochemistry, and Center for Genomics and Proteomics, Brown University, Providence, Rhode Island, United States of America; 6 Institute for Brain and Neural Systems, Brown University, Providence, Rhode Island, United States of America; University of Hong Kong, China

## Abstract

**Background:**

The c-Myc transcription factor is a master regulator and integrates cell proliferation, cell growth and metabolism through activating thousands of target genes. Our identification of direct c-Myc target genes by chromatin immunoprecipitation (ChIP) coupled with pair-end ditag sequencing analysis (ChIP-PET) revealed that nucleotide metabolic genes are enriched among c-Myc targets, but the role of Myc in regulating nucleotide metabolic genes has not been comprehensively delineated.

**Methodology/Principal Findings:**

Here, we report that the majority of genes in human purine and pyrimidine biosynthesis pathway were induced and directly bound by c-Myc in the P493-6 human Burkitt's lymphoma model cell line. The majority of these genes were also responsive to the ligand-activated Myc-estrogen receptor fusion protein, Myc-ER, in a Myc null rat fibroblast cell line, HO.15 MYC-ER. Furthermore, these targets are also responsive to Myc activation in transgenic mouse livers in vivo. To determine the functional significance of c-Myc regulation of nucleotide metabolism, we sought to determine the effect of loss of function of direct Myc targets inosine monophosphate dehydrogenases (IMPDH1 and IMPDH2) on c-Myc-induced cell growth and proliferation. In this regard, we used a specific IMPDH inhibitor mycophenolic acid (MPA) and found that MPA dramatically inhibits c-Myc-induced P493-6 cell proliferation through S-phase arrest and apoptosis.

**Conclusions/Significance:**

Taken together, these results demonstrate the direct induction of nucleotide metabolic genes by c-Myc in multiple systems. Our finding of an S-phase arrest in cells with diminished IMPDH activity suggests that nucleotide pool balance is essential for c-Myc's orchestration of DNA replication, such that uncoupling of these two processes create DNA replication stress and apoptosis.

## Introduction

MYC, which encodes the c-Myc (herein termed Myc) transcription factor, is frequently altered in human cancers [1]–[3]. A compilation of Myc regulated genes and studies on alterations of MYC in human cancers are available on-line at www.myccancergene.org [4]. This compilation highlights the critical role of MYC in human cancers and the importance of Myc target genes in mediating its oncogenic activity. The causal role of MYC in tumorigenesis is underscored by the tumors arising from its ectopic expression in various tissues of transgenic mice [5]–[9]. Subsequent to initial studies demonstrating that forced expression of MYC in lymphoid tissues resulted in lymphoid hyperplasia and lymphomas, virtually all other studies of constitutive or inducible MYC in tissues from skin to liver resulted in neoplastic transformation of the targeted tissue. Studies with these models have also revealed that MYC mediated tumorigenesis includes the concurrent activation of cell growth and proliferation along with inactivation of apoptotic pathways in a tissue specific fashion [8], [9].

The Myc protein heterodimerizes with Max to bind DNA, stimulate and repress transcription[10]–[12]. The Myc-Max network is quite complex, particularly since Max also interacts with six other proteins including Mxd1, Mxd2, Mxd3, Mxd4, Mnt, and Mga[13]–[15]. Many of these non-Myc heterodimers could counter the function of Myc. These findings paint an emerging picture of Myc as an oncogenic transcription factor that is involved in a complicated Max-associated network of proteins to regulate gene expression. Recent findings, however, indicate that Myc also has non-transcriptional functions as well as a role in DNA replication [16]. Specifically, the N-terminal domain of Myc stimulates mRNA cap methylation in the absence of the Myc DNA binding domain[17], [18].

The biological properties of MYC in cell culture and in vivo models suggest that Myc plays a pleiotropic role through its target genes in the regulation of cell size and proliferation as well as cellular metabolism and adhesion. In fact, MYC has emerged as a master regulator that couples cell proliferation to metabolism. To date direct and indirect Myc target genes have been implicated in a variety of cellular processes[19]–[21]. Since MYC induces cell proliferation under specific circumstances, a group of Myc target genes-as expected-involves cell cycle regulation[22], [23]. Activation of positive cell cycle regulators, such as CDK4, and suppression of negative cell cycle effectors, such as p21, have been reported[24]–[27]. In particular, CDK4 has been directly implicated downstream of MYC in the skin tumorigenesis through the use of CDK4 gene deletion in mice [28].

MYC has been connected to different metabolic pathways through its regulation of target genes such as *CAD* (nucleotide metabolism) [29], *ODC* (ornithine/spermine metabolism) [30], *LDHA* (glucose metabolism) [31] and *SHMT2* (single carbon metabolism) [32]. Global gene expression analysis now connects MYC with diverse metabolic pathways with an overrepresentation of Myc responsive genes involved in glucose and nucleotide metabolism. Although Myc's regulation of glucose metabolism has been well delineated, the connection between Myc and purine or pyrimidine metabolism is not well understood. It stands to reason, however, that a master regulator such as Myc must somehow couple cell cycle traversal through S-phase with regulation of nucleotide metabolism and energy generation.

Our global mapping of MYC binding sites in a human B cell lymphoma model identified over 2000 binding sites with 668 direct Myc target genes gleaned from accompanying microarray gene expression analysis [33]. Among the direct Myc target genes is a statistically over-represented small set involved in nucleotide metabolism. This observation led us to determine comprehensively whether genes involved in both purine and pyrimidine synthesis pathways are directly regulated by Myc. We observed that virtually all genes involved in nucleotide *de novo* synthesis are up-regulated and bound by Myc. Although many nucleotide metabolic genes are up-regulated by Myc-ER in Myc null rat fibroblasts, only phosphoribosyl pyrophosphate amidotransferase (PPAT) and dihydroorotate dehydrogenase (DHODH) survived the criterion of cycloheximide treatment, which would prevent indirect activation of targets. Cycloheximide, however, was found to be very problematic due to the noise induced by cycloheximide treatment alone.

To determine the effect of loss of function of target genes involved in nucleotide biosynthesis on MYC-responsive B cell proliferation, we used a specific pharmacological inhibitor of inosine monophosphate dehydrogenase (IMPDH), mycophenolic acid (MPA), and found that inhibition of IMPDH caused DNA replication stress, apoptosis and DNA replication arrest in B cells with high ectopic MYC expression. This arrest was rescued by a downstream metabolite precursor, guanosine, suggesting that maintenance of a balanced nucleotide pool is essential for proper DNA synthesis and cell proliferation. Our studies demonstrate a link between MYC, a master regulator of cell growth and proliferation, and the regulation of nucleotide biosynthesis genes.

## Materials and Methods

### Cell culture

Human Burkitt's lymphoma cell line P493-6, carrying an inducible MYC repression system, was a generous gift of D. Eick (Institute for Clinical Molecular Biology and Tumor Genetics, GSF-Research Centre, Munich, Germany). Cells were cultured in RPMI 1640, 10% fetal bovine serum, and 1% penicillin/streptomycin. Tetracycline (Sigma) treatment at 0.1 µg/ml for 48 hrs was used to repress the ectopic c-*MYC* expression. Ramos cells were obtained from ATCC and cultured in RPMI 1640, 10% fetal bovine serum, and 1% penicillin/streptomycin. For mycophenolic acid (Sigma, dissolved in methanol) and guanosine (sigma, dissolved in 1M acetic acid) treatment, cells were placed in RPMI medium containing the indicated reagents. For growth rate experiments, colorimetric microplate assay Cell Counting Kit-8 (Dojindo) was used according to the manufacturer's instructions. In every 96 well, 15000 Ramos cells or 25000 P493 cells were plated. At 48hr time point, another 100 µl of fresh medium with designated treatment was added. Tetrazolium salt was added to achieve a final concentration of 10% at different time points. Absorbance was measured at 450 nm after 3.3 hour incubation. All experiments were performed in triplicates.

HO.15 MYC-ER, a MYC -/- rat fibroblast cell line overexpressing the MYC-ER construct was maintained in DMEM and 10% fetal bovine serum. These cells were previously described [34], [35]. In the MYC-ER induction experiment, HO.15 MYC-ER cells are cultured in either regular medium (DMEM+10%FBS) or serum starvation (DMEM+0.25% FBS) for 48 hrs before the 4-hydroxytamoxifen stimuli. Cells are treated with cycloheximide (10 µM) or DMSO vehicle ctrl for 30 minutes before the 4-hydroxytamoxifen (250nM) or ETOH vehicle ctrl was added to the tissue culture medium. Cells were collected for RNA extraction at time points: 0 hr, 2 hr, 4 hr and 10 hr after the addition of 4-hydroxytamoxifen. [36]

### Western blot analysis

For immunoblot analysis, cells were lysed in incomplete Laemmli buffer (lacking β-mercaptoethanol and dye) and subsequently boiled at 95°C for 5 minutes before quantification. Protein concentration was measured by using a bicinchoninic acid kit (Pierce). Samples of 15 to 20 micrograms of protein with β-mercaptoethanol and dye were loaded on the Nupage ® Bis-Tris 4-12% gradient gel (Invitrogen) and transferred to a nitrocellulose membrane. The protein expression of c-Myc was detected by 9E10 antibody produced from ascites with the original hybridoma clone.

### In vivo analysis of nucleotide de novo synthesis in murine neonatal hepatocarcinoma

The conditional c-MYC-transgenic neonatal hepatocellular carcinoma model was previously described[37]. The animals were maintained on regular drinking water or water supplemented with 0.01% doxycycline when the female LAP-tTA and the male TRE-Myc transgenic mice were crossed and maintained as it was after the babies were born till 11 days to collect the liver tissue.

### Chromatin immunoprecipitation

Untreated P493-6 cells, cells subjected to Tetracycline treatment for 48 hours, and cells withdrawn from Tetracycline treatment at different time points (6 hr, 10 hr, and 26 hrs) were used for chromatin immunoprecipitation (ChIP) assays. Chromatin was immunoprecipitated with the rabbit polyclonal c-MYC (sc-764; Santa Cruz Biotechnology) and human hepatocyte growth factor (sc-7949; Santa Cruz) antibodies as previously described[4], [33], [38]. Total input controls were collected from the supernatant of the “no antibody” control.

### Double thymidine block

Synchronization of P493-6 cells was achieved by incubating cells with 2mM thymidine for 12 hours, washing and incubating cells with normal growth medium for another 12 hours, and treating cells with a second time thymidine for 12 hours[39]–[42]. After washing, cells were subsequently treated with either vehicle or the MPA and guanosine combination as indicated. Cells were collected for cell cycle profiles at different time points (4 h, 8 h, and 12 h) after the double thymidine block. Cell cycle profiles were analyzed by using propidium iodide (PI) staining[43].

### Flow cytometry

For PI staining, 1×10^6^cells were re-suspended and fixed with 70% ethanol at −20°C for at least an hour. Before analysis, cells were re-suspended in PBS containing 100 µg/ml RNaseA (Roche) and 50 µg/ml PI (Sigma) for at least an hour.

For BrdU uptake assay, FITC BrdU Flow Kit (BD Pharmingen) was used. Approximately 1×10^6^cells were pulsed with 10 µM BrdU in the regular medium (RPMI1640+10%FBS+1%Pen/strep) for 30minutes before subsequent fixation and staining according to manufacturer's instructions.

For apoptosis detection, Annexin V-PE Apoptosis Detection kit I (BD Pharmingen) was used according to the manufacturer's instructions. Cells were filtered and then analyzed using a Becton Dickinson FACScan or FACSCalibur flow cytometer.

### Real-time PCR

Quantitation of ChIP fragments was performed as described [33] using the Power SYBR Green PCR Master Mix (ABI) according to the manufacturer's instructions with the ABI 7700 sequence detection system or ABI 7500 Realtime PCR system. Specific primers were designed using the Primer Express software (**supplemental Table S3**). Known quantities of 10-fold dilutions of total input DNA were used to generate standard curves for each primer pair. Relative amounts of each ChIP sample (expressed as the percentage of total input) were determined in the linear range according to their *C_T_* value. Dissociation curves were used to verify the correct PCR product for each primer pairs.

Total RNA was extracted from P493-6 cells with RNeasy kit (QIAGEN) or extracted from mouse liver tissue with Trizol (Invitrogen). cDNA was obtained by using Taqman reverse transcription reagents. To examine the expression level of the nucleotide de novo synthesis, primers were designed to cross exon-exon junctions (**supplemental Table S2**) and the Power SYBR Green PCR Master Mix (ABI) was used. Human c-MYC was detected with primers (Forward: TCAAGAGGTGCCACGTCTCC, Reverse: TCTTGGCAGCAGGATAGTCCTT) and probe (CAGCACAACTACGCAGCGCCT) with the TaqMan Universal PCR Master Mix(ABI). A predeveloped probe and primers specific to 18S rRNA levels were used for normalization. All PCRs were performed in triplicates.

### HO.15 MYC-ER microarray

#### Cell culture for array analysis and expression profiling

The cell line HOMycER12 was used [34], [35]. This cell line was derived from the c-myc-/- cell line HO15.19, and expresses a cDNA encoding a c-Myc -estrogen receptor (ER) fusion protein. MYC-ER cells were kept in a constant state of proliferation and the MYC-ER protein was activated by addition of 4-hydroxytamoxifen (OHT) to the medium, as described [35]. Cells were collected at the following time (t) points after addition of OHT: 1, 2, 3, 4, 5, 6, 8, 10, 12, 16, 20 and 24 hours. A sample was also collected at the time of OHT addition (t = 0 h). Negative controls were collected at 8 h, 16 h and 24 h following mock addition of OHT (ethanol vehicle). Total RNA was harvested from each sample, and was hybridized to GeneChip (r) Rat Exon 1.0 ST Arrays (Affymetrix). The entire process was repeated to generate three independent biological replicates.

#### Probeset filtering and normalization

The PLIER algorithm from the Affymetrix Power Tools (APT) software package was used to generate gene and exon expression scores. Probes were filtered and remapped according to the ENTREZG tables provided by the Microarray Lab of the Molecular and Behavioral Neurosience Institute, University of Michigan[44]. Probes were remapped by blasting their sequences onto the rn4 genome and removing probes mapping to multiple locations in the genome. Genes were redefined as sets of at least three probes mapping to the same Entrez gene designation. These data will be deposited in the National Center for Biotechnology Information Gene expression Omnibus (GEO).

#### Statistical Analysis of microarray data

We first excluded from the analysis genes whose PLIER scores, averaged over the three biological replicates, did not exceed a value of 48 for all time points, (the 1st quartile of all the average scores over all replicates, which we considered below the noise threshold). We then applied a 3-way ANOVA with the following factors: time, treatment, and experiment (each biological replicate and its associated controls were considered as one independent experiment). The experiment factor was included since we have evidence that scores from the same experiment are correlated. For each gene, the time factor p value was calculated based on differential expression between any time point (t “greater or equal to” 1 h) and the t = 0 time point; the treatment factor p value was calculated based on differential expression between treated and control samples. The p-value threshold was set using a Bonferroni correction, in which the p-value cutoff (0.05) is divided by the number of genes (21,334). After this analysis, 1,612 genes were deemed significantly differentially expressed following c-MYC induction.

## Results

### Myc upregulates the expression of nucleotide *de novo* genes *in vitro*

**Figure 1:**
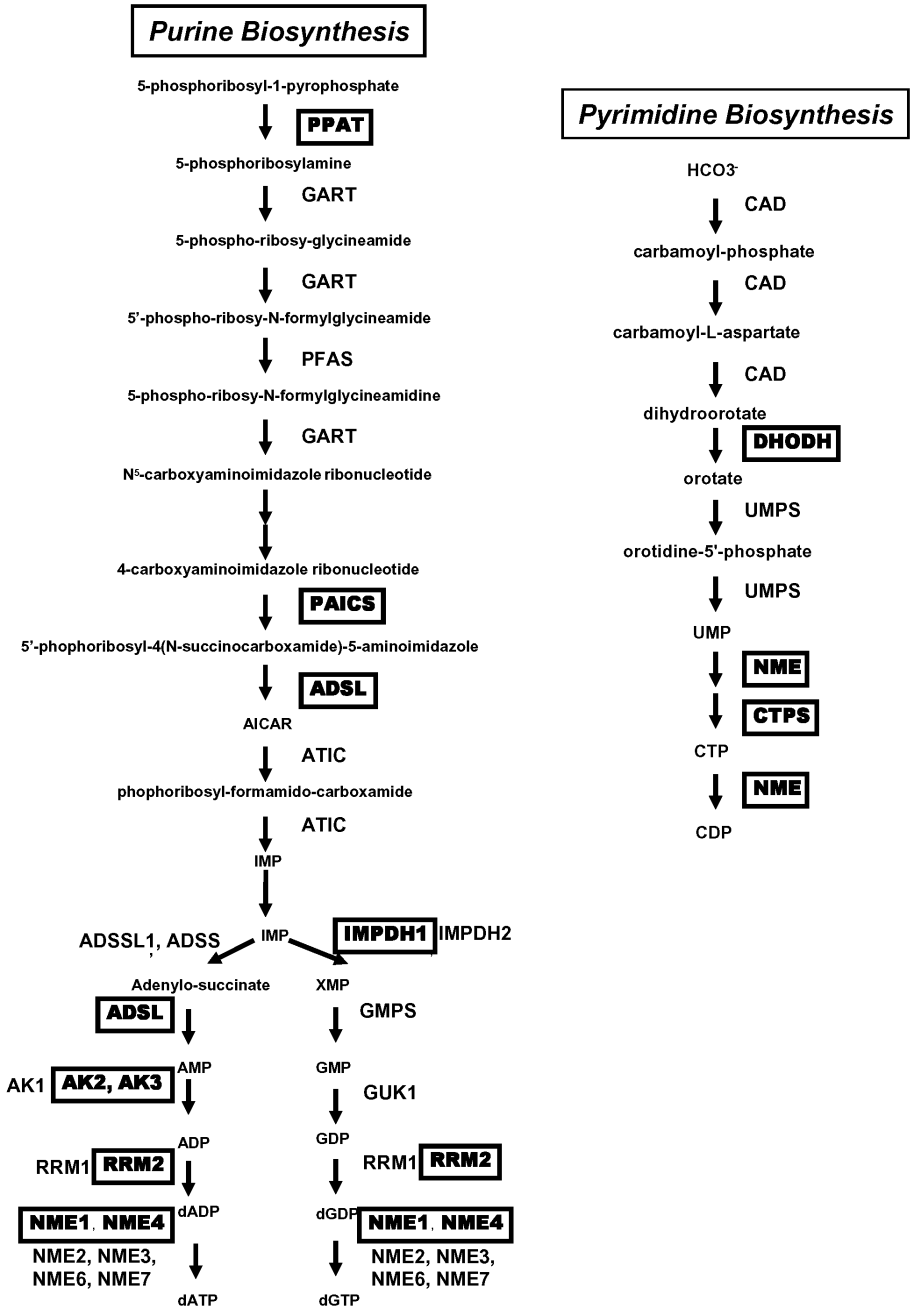
Purine and pyrimidine biosynthesis pathways. The inset provides a legend of nucleotide metabolism genes with direct MYC binding found by ChIP-PET in P493-6 model [33].

From our previous genome-wide mapping of Myc binding sites via coupling chromatin immunoprecipitation with pair-end ditag sequencing analysis (ChIP-PET) in P493-6 cells [33], we found that among sets of genes bound by Myc, nucleic acid metabolism is one of the most significantly enriched functional class based on Gene Ontology categorization through the PANTHER database (panther.appliedbiosystems.com). The P493-6 cell line, an EBV immortalized B lymphocyte engineered with a tetracycline (Tet)-repressible c-MYC expression vector, is considered as an *in vitro* Burkitt's lymphoma model [45]. P493-6 cells express high levels of Myc in the absence of tetracycline with a Burkitt's lymphoma-like cytologic morphology. Within the purine and pyrimidine de novo synthesis pathway, 11 genes were bound directly by Myc in the ChIP-PET study **(** **Figure 1** **).** Genes encoding almost every step involved in the purine and pyrimidine de novo synthesis pathway are up-regulated after MYC induction in the P493-6 system as determined by 5 microarray experiments on biological duplicates or triplicates. The majority of the nucleotide biosynthesis genes were also found to have canonical E boxes within either their promoters or first introns **(** **supplemental Table S1** **).** Altogether, these findings strongly indicate that MYC could bind and presumably directly activate both purine and pyrimidine synthesis genes.

To delineate if Myc directly regulates the nucleotide de novo biosynthetic genes, the expression levels of these genes in response to Myc were examined and ChIP assays were performed. We used quantitative real-time PCR (qPCR) to assay mRNA levels and ChIP products in P493-6 cells.

**Figure 2:**
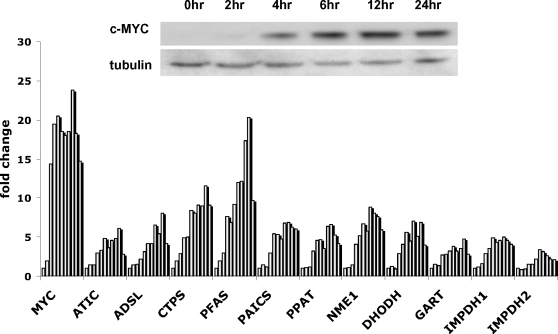
Nucleotide biosynthetic genes are responsive to MYC induction in human B cells *in vitro.* The expression of the nucleotide synthetic genes is temporally responsive to Myc induction. The inset shows an immunoblot of Myc expression in P493-6 cells withdrawn from tetracycline. Bar graphs represent mRNA expression of each indicated nucleotide synthetic gene relative to 18S rRNA control as determined by triplicate real-time PCR reactions (with less than 5% error from the mean) in P493-6 cells with a time course after tetracycline withdrawal (0, 2, 4, 6, 8, 10, 12, 16, 24, 32 and 48hrs after tetracycline removal). MYC was used as a positive control. MAX mRNA level was not found to be affected by MYC induction in P493 cells (not shown).

The expression of 11 genes in P493-6 cells was examined separately at different time points after tetracycline withdrawal to elucidate the kinetics of nucleotide biosynthetic gene induction by MYC. While Myc protein level increases by 4 hours (**Figure 2**, **inset**), qPCR assays showed significant induction of all 11 genes examined between 6 to 12 hours after removal of tetracycline (**Figure 2**). The observations of the temporal response of nucleotide biosynthesis genes to Myc support a role for Myc in regulating nucleotide metabolism in the B cell model.

### c-MYC upregulates the expression of nucleotide biosynthetic genes *in vivo*

**Figure 3:**
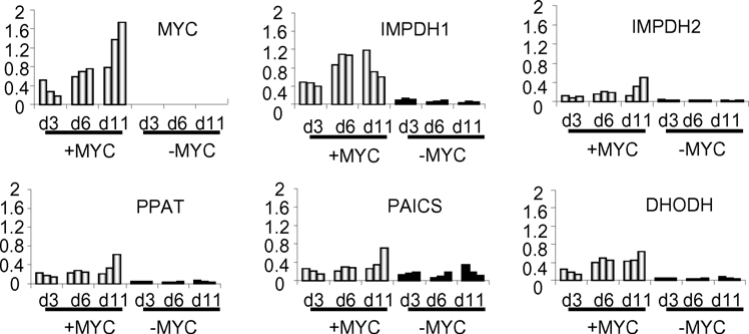
The expression of selected key nucleotide synthesis genes is responsive to MYC in hepatocytes *in vivo*. LAP-tTA X TRE-Myc transgenic mouse conditionally expresses MYC proto-oncogene in hepatocytes. Male littermates withdrawn from doxycycline at birth overexpressed MYC. Littermates withdrawn from doxycycline remained tumor-free up to day 3 (d3) but all developed liver tumors by day 11 (d11). Newborn mice continuously treated with doxycycline remained free of disease. In both groups (with or without doxycycline treatment), 3 mice were sacrificed at each time point (d3, d6, and d11) and the RNA extract from each liver sample was subjected to real-time PCR analysis for the expression of key genes. Bar graphs represent mRNA expression of human MYC and each nucleotide biosynthetic enzyme gene in the pathway relative to 18S rRNA control.

Since most of the nucleotide biosynthetic gene expression level is tightly coupled to Myc expression *in vitro* in the human B lymphocyte model, we sought to characterize whether selected nucleotide metabolic genes are also induced by Myc *in vivo*. We used a murine inducible MYC transgenic hepatocarcinoma model that provides ample tissue for gene expression analysis. The TRE-LAP MYC transgenic mice over-express human MYC in liver cells upon removal of doxycycline from the drinking water resulting in hepatocellular carcinoma (HCC) in neonates from pregnant females [37]. While neonates up to 3 days do not develop HCC even with high MYC expression, neonates at 6 days of age or older demonstrated liver cancer cells that effaced the entire liver. By neonatal day 11, all animals have HCC. In this model, ectopic MYC expression is induced nearly 10-fold in non-tumorous livers above doxycycline-treated controls. The expression of MYC is further increased in tumor cells by an additional 10-fold through an unknown mechanism [37]. We sought to study the expression of PPAT, PAICS, IMPDH1, IMPDH2 and DHODH in these livers. We were interested in PPAT and PAICS because they share a bi-directional promoter and are both key enzymes in purine synthesis (**Figure 1**). For IMPDH and DHODH, there are clinically available small molecules that could specifically inhibit their function separately. We compared the expression of these selected nucleotide biosynthesis genes by qPCR at neonatal day 3, 6 and 11 in livers from 3 animals in each group treated (-MYC) or untreated (+MYC) with doxycycline (**Figure 3**). Note that in the absence of tumor formation at day 3, the expression of PPAT, IMPDH1, IMPDH2, DHODH but not PAICS are all statistically significantly elevated with MYC induction. The expression levels were further increased in the tumors at neonatal day 11. These observations indicate that, all the genes examined except PAICS are all responsive to MYC in vivo.

### Myc directly binds to the regulatory regions of nucleotide biosynthetic genes in the human P493 B cells

**Figure 4:**
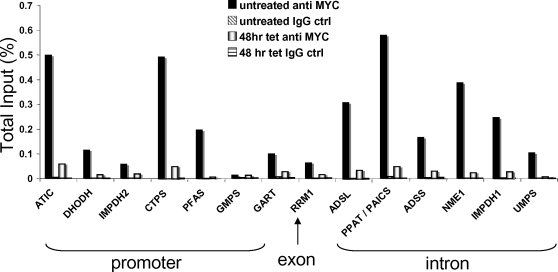
MYC binds to the regulatory regions of *de novo* nucleotide synthetic genes in B cells. Chromatin immunoprecipitation assay demonstrates direct Myc binding to the nucleotide synthesis genes in P493-6 cells. Chromatin from P493-6 cells with or without tetracycline treatment for 48 hours were precipitated with either anti-Myc or control IgG as indicated. The promoter or intronic sequence of each gene was quantitatively amplified by real-time PCR. Bar graphs represent Myc binding to chromatin regions that contain promoters (ATIC, CTPS, DHODH, GART, GMPS, IMPDH2, and PFAS), exons (RRM1) or introns (ADSL, ADSS, IMPDH1, NME1, PPAT/PAICS, and UMPS). Note that PPAT and PAICS are shown together because they share a bidirectional promoter. Significant binding is >0.02% total input.

We determined the binding of Myc to the promoters or intronic sequences of specific nucleotide metabolic genes to provide additional evidence for their direct regulation by Myc. As shown in **Figure 4**, Myc bound most of these genes except GMPS. The binding was dependent on the induction of Myc protein. The primers used in the ChIP experiment amplified regions that are proximal to canonical E boxes or PET-clusters identified by ChIP-PET study. For genes without canonical E box or PET-cluster, primers were designed to cover the promoter region. The binding of Myc to most nucleotide metabolic genes strongly supports the hypothesis that Myc directly activates nucleotide biosynthesis.

### The MYC-ER system validates PPAT and DHODH as direct c-MYC target genes in rat fibroblasts

**Figure 5:**
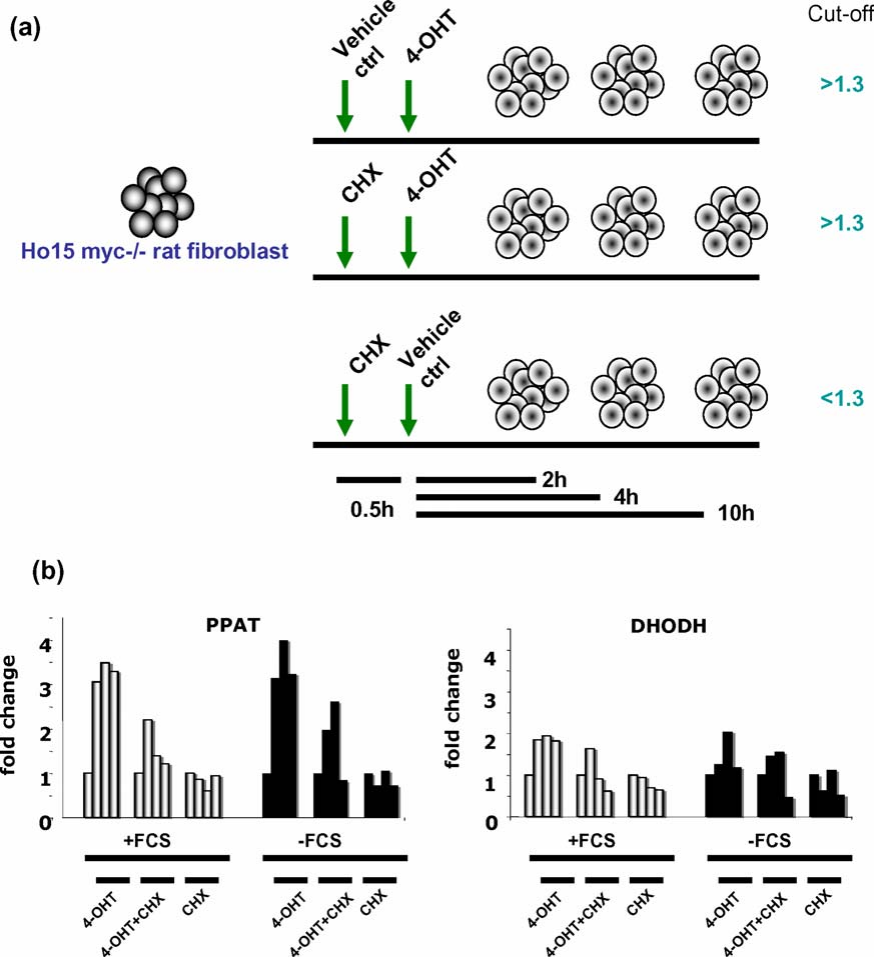
PPAT and DHODH are direct c-MYC target genes in the HO.15 MYC-ER system. (A) The experiment design was shown in the diagram. We set the following criteria to identify direct MYC target genes: 1) A>1.3 fold induction of expression by 4-OHT with or without cycloheximide; 2) A<1.3 fold induction of expression by cycloheximide alone. (B) PPAT and DHODH fulfilled the criteria in the majority of the experiments. Bar graphs represent mRNA expression of either PPAT or DHODH relative to 18S rRNA control as determined by real-time PCR in HO.15 MYC-ER cells with regular medium (+FCS; light bars represent the expression at 0, 2, 4,10 hr) or with serum starvation (-FCS; black bars represent the expression at 0, 2, 4,10 hr). One representative experiment is shown for each group from 4 biologically independent experiments.

The Myc-estrogen receptor hormone binding domain (Myc-ER) fusion protein system is another important model for the identification of direct Myc target genes. In the absence of estrogenic compounds, the Myc-ER protein is bound by HSP90 in the cytoplasm. Upon binding estrogenic compounds such as 4-hydroxytamoxifen, the chimeric protein disengages from HSP90 and translocates to the nucleus, dimerizes with Max and activates transcription even in the presence of cycloheximide, which blocks protein synthesis. Hence, direct Myc target genes are considered to be those that are responsive to Myc-ER in the presence of cycloheximide. This system is confounded by changes in the expression of specific genes induced by cycloheximide alone. In this regard, we arbitrarily consider direct Myc targets in this system to be induced at least 1.3 fold by Myc-ER in the presence of cycloheximide but no more than 1.3 fold by cycloheximide alone **(** **Figure 5a** **)**.

Using the Myc null rat fibroblast (HO.15) expressing MYC-ER [35], we found that cycloheximide alone perturbed the expression of many nucleotide metabolic genes. Nonetheless, among 4 biologically independent experiments, 2 with regular culture medium and 2 deprived of serum to eliminate cell proliferative effects of Myc induction, PPAT and DHODH behaved as direct Myc target genes **(** **Figure 5b** **).** All the other genes examined, except GUK1, responded to the MYC-ER induction early in the time course (2–4 hrs after the estrogenic compound treatment). The expression of most of the genes were affected by cycloheximide treatment alone **(** **Table 1** **and** **supplemental Figures S1** **and** **S2** **)** [**35**]. Hence, the noise imparted by cycloheximide alone profoundly limits the use of this system to identify direct target genes in the nucleotide biosynthesis pathway.

**Table 1:** Response of nucleotide biosynthetic enzyme genes to MYC in HO15.MYC-ER cells

Gene Symbol	Induction time point	Fold change	Direct/Inconclusive
ADSL	2hr	1.9–2.5	I
AK2	2hr	1.8–2.6	I
CAD	2hr	3.4–6.8	I
CTPS	2hr	2.7–4.4	I
DHODH	2hr	1.9–5.2	D
GART	2hr	2.3–8.1	I
GMPS	2hr	2.4–6.6	I
GUK1	no	NC	I
IMPDH1	2hr	1.9–5.9	I
IMPDH2	2hr–4hr	1.4–2.7	I
PAICS	2hr	1.3–4.0	I
PFAS	2hr	2.3–3.8	I
PPAT	2hr	3.5–11.4	D

Note: Induction time point was determined by the time point when the induction >1.3 fold in the majority of the 4 independent experiments. Direct/Inconclusive was determined by the arbitrary criteria: 4-OHT±CHX>1.3, CHX<1.3, D: direct, I: inconclusive

### Myc regulates the *de novo* purine and pyrimidine synthetic genes in multiple biological systems

**Table 2** summarizes the results from experiments we performed to delineate the direct regulation of the nucleotide biosynthetic genes by Myc. We examined the response of nucleotide biosynthetic gene expression to Myc *in vitro* in the B cell model and rat fibroblasts, and *in vivo* in the transgenic mouse model. ChIP of Myc binding to nucleotide biosynthetic genes performed in B cells and gene expression determined in B cells, MYC-ER rat fibroblasts or mouse livers indicate that the vast majority of nucleotide biosynthetic genes are probably direct Myc targets. PPAT, catalyzing the first step of purine synthesis, and DHODH, an enzyme generating uridine in the middle of the pyrimidine synthesis pathway, were validated as direct c-MYC target genes by all criteria. The other genes listed could still be directly regulated by MYC but failed to survive the criterion set by the MYC-ER system, which is confounded by noise imparted by cycloheximide itself.

**Table 2:** Responses of genes encoding nucleotide biosynthesis enzymes to MYC

	ADSL	ADSS	AK2	ATIC	CAD	CTPS	DHODH	GART	GMPS	IMPDH1	IMPDH2	NME	PAICS	PFAS	PPAT	RRM	UMPS
A	V	V		V		V	V	V	V	V	V	V	V	V	V	V	V
B							V			V	V		X		V		
C	V	V		V		V	V	V	X	V	V	V	V	V	V	V	V
D	X		X		X	X	V	X	X	X	X		X	X	V		

A: MYC responsiveness in human B cell in vitro,

B: MYC responsiveness in mouse hepatocyte in vivo,

C: MYC direct binding in human B cell in vitro,

D: HO.15 MYC-ER system, from 4 independent biological experiments

V = positive, X = negative or inconclusive, Empty = not checked

The mRNA levels of IMPDH1 and IMPDH2, the rate-limiting enzyme in purine *de novo* synthesis, increased with MYC induction both in vitro and in vivo. Furthermore the promoter regions of both genes are bound directly by Myc, suggesting that they are direct Myc targets which did not respond accordingly in the MYC-ER system (**supplemental Figure S3**). The putative direct regulation of IMPDH2 by Myc is further underscored by the highly statistically significant co-expression of MYC and IMPDH2 (Pearson correlation coefficient >0.85) in microarray experiments among 73 human tissues or cells and 6 human cell lines (http://symatlas.gnf.org/SymAtlas).

### Loss of IMPDH activity inhibits cell proliferation and survival of P493 B cells

**Figure 6:**
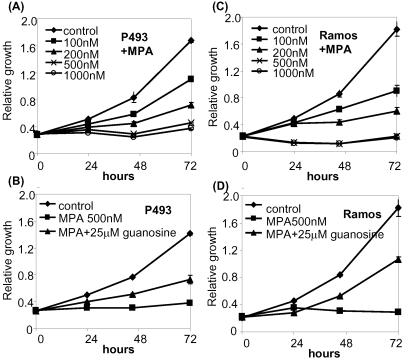
Mycophenolic acid (MPA) inhibits proliferation in P493-6 cells (left panels) and Ramos human Burkitt's lymphoma cell line (right panels). Cell proliferation was analyzed by using colorimetric microplate assay in which tetrazolium salt was bioreduced to orange product. Y-axis represents the absorbance measurement at 450 nm. Error bars represent standard deviations derived from three independent measurements. (A) Growth rates of P493-6 cells at different concentrations of MPA. (B) Growth rates of MPA-treated P493-6 cells with guanosine supplement. (C) Growth rates of Ramos cells at different concentration of MPA. (D) Growth rates of MPA-treated Ramos cells with guanosine supplement.

Because many nucleotide metabolic genes appear to be directly regulated by Myc, we sought to determine the effects of loss of function of a specific enzyme, IMPDH, in purine metabolism on Myc-mediated cell proliferation and growth. In particular, we wish to delineate whether inhibition of IMPDH in high MYC expressing cells would simply deprive cells of guanosine and cause a G1-S checkpoint arrest or cause S-phase stress with apoptosis as an exit strategy for the perturbed cells. To determine if IMPDH is essential for MYC stimulation of cell proliferation, we used mycophenolic acid (MPA), the active metabolite of the well-known clinical immunosuppressant Cellcept and specific inhibitor of IMPDH1 and IMPDH2 [46]. MPA inhibited P493-6 cell population growth in a dose-dependent manner with dose ranges that are clinically achievable (**Figure 6A**). We also found that guanosine, which is converted first to guanine and then to GMP through HGPRT, could partly rescue the proliferative inhibitory effect of MPA (**Figure 6B**) [46]. We also studied the Ramos human Burkitt lymphoma cell line, and found that it is similarly inhibited by MPA (**Figure 6C**) and also rescued by low doses of guanosine (**Figure 6D**). Intriguingly, high doses of guanosine inhibit cell proliferation that is associated with apoptosis and cell cycle arrest (data not shown). Hence, guanosine's own growth inhibitory effects at high concentrations confound its ability to rescue MPA inhibition. As such, we presumably could not use a sufficiently high concentration of guanosine to replenish the deficient state caused by MPA.

**Figure 7:**
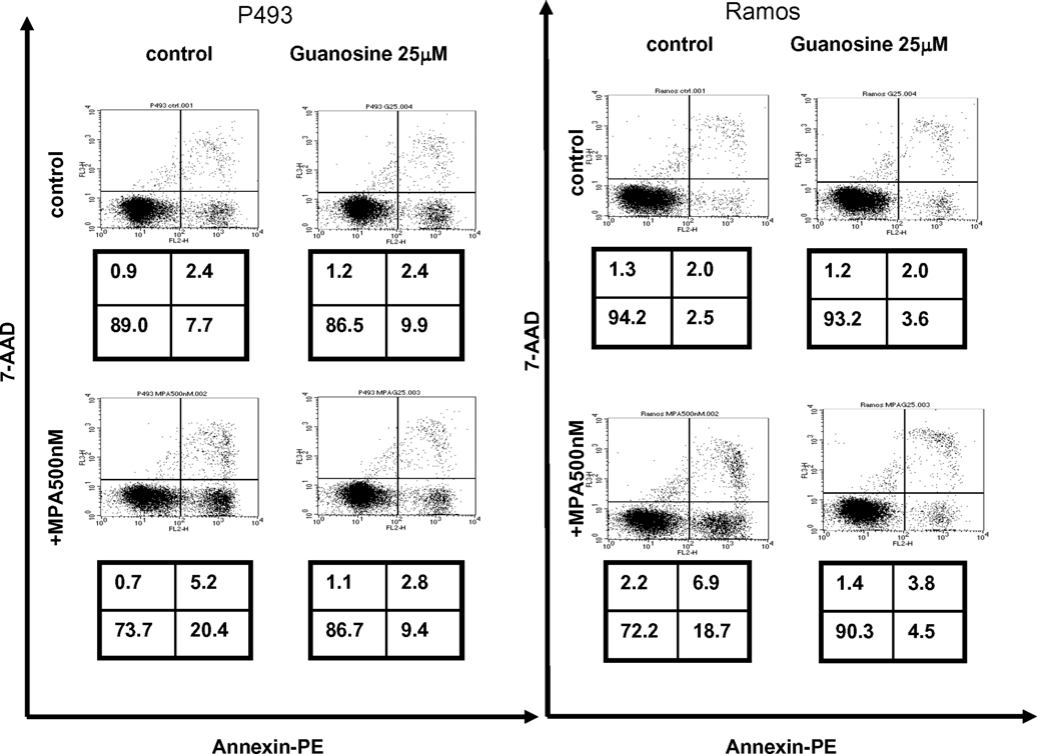
Mycophenolic acid (MPA) induces apoptosis in P493-6 and Ramos cells. Cells with designated treatments were subjected to annexin V staining with 7-AAD and flow cytometry after 48 hours. Cells in the lower right quadrant, which are annexin-positive but 7-AAD-negative, represent the apoptotic population. Necrotic or late apoptotic cells are 7-AAD-positive. The number indicates the percentage of the cells in each quadrant.

**Figure 8:**
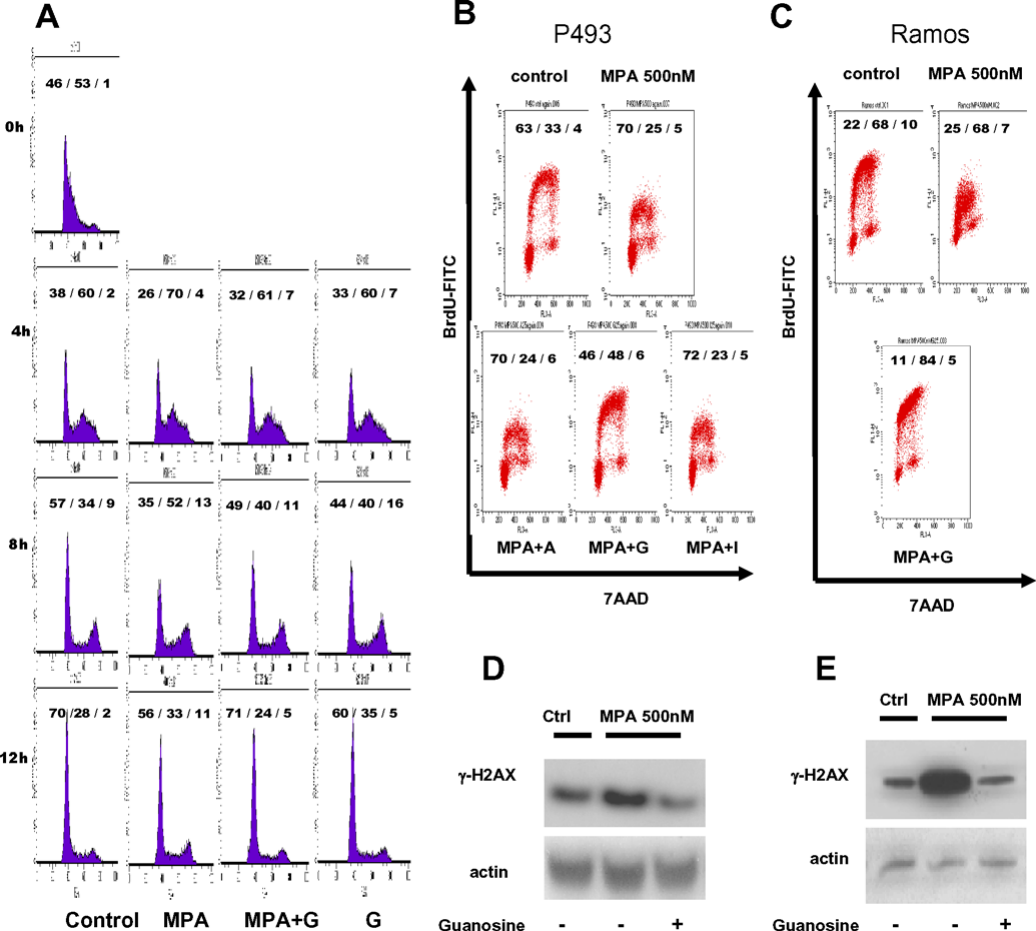
Mycophenolic acid (MPA) slows DNA replication in P493-6 cells. (A) P493-6 cells were synchronized with double thymidine block. Cells were incubated with 2mM thymidine for 12 hours, washed and incubated with normal growth medium for another 12 hours, and underwent a second thymidine treatment for 12 hours. After washing, cells were subsequently treated with either methanol vehicle or MPA and guanosine as indicated. Cells were collected for cell cycle profile analysis at different time points (4 h, 8 h, and 12 h) after release from double thymidine block. Cell cycle profiles were analyzed by flow cytometry using PI staining. Numbers indicate the percentage of the cells in each cell cycle phase (G1/S/G2M). (B) DNA replication and cell cycle profile were examined by BrdU uptake assay. P493-6 cells were pulsed with BrdU for 30 minutes before fixation and subsequent staining. Anti-BrdU FITC antibody was used for measuring the BrdU incorporation. DNA content was assessed with 7-AAD staining. MPA-treated cells were incubated concurrently with the indicated nucleotide (A = 25 µM adenosine; G = 25 µM guanosine; I = 25 µM inosine) supplement for 48 hrs before subjected to the analysis. (C) BrdU uptake assay in Ramos cells treated with MPA or with both MPA and 25 µM guanosine(G). Cells were processed as described in Panel 8B. (D) Immunoblot demonstrating the expression of γ-H2AX, a marker for DNA double strand breaks, in MPA-treated P493-6 cells. Protein lysates from cells with designated conditions were harvested 48 hrs after guanosine treatment. In the groups receiving both MPA and guanosine supplement, the treatment started concurrently. (E) γ−H2AX expression in MPA-treated Ramos cells with or without 25 µM guanosine.

We next determined whether MPA diminishes P493-6 cell growth through apoptosis. Annexin V staining indicates that MPA triggered moderate apoptosis that could be largely rescued by guanosine (**Figure 7**). Similarly, we observed that the MPA-treated apoptotic Ramos cells were also rescued by guanosine (**Figure 7**). Since MPA-induced apoptosis was almost fully rescued by guanosine, apoptosis is unlikely to account fully for the slowed population doubling with exposure to MPA. Hence, we determined the effects of MPA on the cell cycle. P493-6 cells synchronized by double thymidine block were released in the presence or absence of MPA and cells were harvested at 4, 8, and 12 hours for flow cytometry (**Figure 8A**). Staining of cells for DNA content reveals that MPA treated cells persisted longer in S phase at 4 and 8 hours as compared with control cells or cells treated with MPA but rescued with guanosine. These studies suggest that MPA causes a delay in S phase.

To ascertain the effects of MPA on S phase, P493-6 cells were doubly labeled with BrdU and 7-amino-actinomycin D (7-AAD) for flow cytometry, which provides a measure of DNA synthesis via BrdU incorporation and distribution of cells in the different phases of the cell cycle via DNA content determined by 7-AAD staining. Studies were performed after treatment with MPA with and without guanosine rescue (**Figure 8B**). MPA treated P493-6 cells display a population of S-phase cells that have severely blunted incorporation of BrdU that could be rescued by guanosine, but not by adenosine or inosine (**Figure 8B**). We note in the guanosine-rescued cells, however, a change in the morphology of the BrdU and 7-AAD 2-dimensional flow cytometric dot plot. In particular, the S-phase BrdU arc, which normally curves down toward the G2M fraction of cells, is increased and peaked over the G2M region of the rescued cells. We surmise that while additional dGTP provided by guanosine is sufficient to allow additional incorporation of BrdU, the MPA-treated cells reaching G2M are not able to divide in the time window of the BrdU pulse. The blunted BrdU incorporation with MPA treatment suggests that DNA stress due to limited dGTP results in slowed DNA replication and diminished cell proliferation. We similarly found that the blunting of BrdU incorporation by MPA in Ramos cells could be fully rescued by guanosine (**Figure 8C**). Furthermore, we observed that MPA treatment activated the phosphorylation of H2AX (γH2AX) that could be rescued by guanosine in both P493-6 (**Figure 8D**) and Ramos cells (**Figure 8E**). These observations are compatible with DNA stress induced by MPA depletion of dGTP, particularly at 500nM MPA which caused an S phase arrest or delay. In aggregate, the studies suggest that an ongoing maintenance of nucleotide pools is necessary for normal DNA replication, which could be stressed by imbalance in these pools particularly in Myc-driven cells. These findings also strongly suggest that the upregulation of IMPDH1 and IMPDH2 by MYC is essential for the proper transit of cells through S phase, such that interruption of IMPDH function results in an S phase delay or arrest that is accompanied by evidence of replication stress and apoptosis.

## Discussion

MYC is not only a proto-typical oncogene when activated in cancers, but it is also a master regulator of normal cell growth, cell proliferation and metabolism. In this regard, Myc as a transcriptional regulator has been shown to regulate a number of cellular metabolic functions including its upregulation of glycolytic genes and genes involved in oxidative phosphorylation and mitochondrial biogenesis. The coupling of energy metabolism and the cell cycle machinery by Myc ensures that adequate energy is attained for DNA synthesis. Hence, we sought to determine in this study whether Myc also couples cell cycle progression with regulation of nucleotide metabolism.

A balanced pool of dNTPs is not only required for the fidelity of DNA replication, but a total increase in the pool during S phase is also essential for cell proliferation [47], [48]. Previous studies have shown that regulation of this pool is critical, such that an increase in any component of the pool would lead to misincorporation of nucleotides, resulting in mutagenesis [48]. Although the balance of the dNTP pool is regulated largely by the numerous enzymes catalyzing the inter-conversion of dNTPs, how the cell increases the total dNTP pool in synchrony with the cell cycle and DNA replication machinery is largely unknown [47]. Here, we demonstrate that one of the key regulators of cell cycle, MYC, can also directly activate genes involved in purine and pyrimidine biosynthesis and prepare the cells ready for cell cycle transition.

To identify direct Myc target genes, it is crucial to determine if the target genes are directly bound by Myc and their expression responds to Myc induction. ChIP is the only easily accessible method that provides direct evidence for the *in situ* association of a transcription factor to a specific target gene. We observed that the majority of the genes in the nucleotide biosynthetic pathways are directly bound by Myc. Furthermore the expression of these genes also peaked after the induction of Myc, thereby supporting the direct regulation of purine and pyrimidine biosynthetic genes by Myc.

Another criterion for the identification of direct Myc target genes is the induction of a target gene in the MYC-ER system in the presence of cycloheximide. As discussed above, the inhibition of protein synthesis by cycloheximide is expected to prevent synthesis of a secondary factor that could activate secondary responsive genes. Hence, genes that respond to MYC-ER in the presence of cycloheximide are considered to be direct Myc targets. Although hypothetically appealing as a principle, the MYC-ER system is confounded by the effects of cycloheximide itself on the mRNA levels of specific genes. In this regard, the MYC-ER system is a highly stringent system for the inclusion of direct Myc targets, but its limitation prevents its use to eliminate genes as direct targets of Myc. In particular, it is possible that a feed-forward loop could be driving a direct Myc target such that Myc induces a hypothetical transcription factor X, which together with Myc are both required to activate a direct Myc target gene. The use of cycloheximide would prevent the induction of factor X and hence blocks the induction of a feed-forward loop direct Myc target. In our experience, the use of cycloheximide is associated with significant noise in the mRNA levels of specific genes in the nucleotide metabolic pathways. Well-known direct Myc targets such as the CAD (carbamoyl-phosphate synthetase 2, aspartate transcarbamylase, and dihydroorotase) did not behave as a direct Myc target in our current study[29]. Hence, the lack of the typical pattern of MYC-ER responsiveness with or without cycloheximide should not be used to exclude genes as direct c-MYC targets. Notwithstanding these caveats, PPAT and DHODH survived the MYC-ER criterion and were found to be bona fide direct Myc targets that are involved in purine and pyrimidine biosynthesis.

Intriguingly, MYC was found to directly activate the expression of SHMT1, and SHMT2, which are enzymes involved in single carbon metabolism and are essential for dNTP synthesis [32]. SHMT1 and SHMT2 donate single carbons to tetrahydrofolate for the methylation of dUMP to dTMP by thymidylate synthase, which was demonstrated to be a direct MYC target [49]. SHMT2 was previously discovered as a gene that could partially rescue the diminished growth of rat fibroblasts lacking both copies of c-MYC [32]. Our work indicates that MYC globally activates nucleotide biosynthesis by directly transactivating virtually all genes involved in purine and pyrimidine synthesis. In this fashion, the upregulation of cell cycle genes, such as cyclin D2 and CDK4, by MYC is coupled with an increase in dNTP nucleotide pool through direct activation of nucleotide biosynthetic genes. Nucleotide metabolic genes are hence not just ‘housekeeping’ genes.

The coupling of cell cycle traversal with nucleotide biosynthesis is likely to involve the E2F family members since ribonucleotide reductase is a known target of E2F1 [50], [51]. Moreover, our bioinformatics analysis of Myc binding sites identified by ChIP-PET revealed that the consensus binding site for E2F is highly statistically enriched in DNA fragments associated with Myc [33]. Analysis of target genes with both Myc and E2F consensus sites reveal a group that are directly involved in DNA replication, such as MCM3, MCM4 and CDC6. In addition, analysis of the nucleotide metabolic gene promoters also reveals an enrichment of E2F sites. It is notable that Myc can induce E2F1 as a direct target, and in this regard Myc and E2F1 could participate in a putative feed-forward loop with nucleotide metabolic genes as direct targets of both transcription factors. We surmise that the orchestration between the cyclin-CDK axis, the DNA replication machinery and nucleotide pool regulation involve coordinating transcriptional regulation by MYC and E2F; however, the role of E2F family members in this context is beyond the scope of the current work.

Nucleotide biosynthesis has been a cornerstone target for cancer chemotherapy that has contributed to the remarkable responses of many cancers including lymphoid malignancies. In fact, some of the more profound immunosuppressants, such as MPA and leflunomide, are inhibitors of nucleotide biosynthesis that is clearly required for normal lymphocytic immune function. We sought to determine the influence of MPA, which inhibits IMPDH function and hence reduction of GMP, on the proliferation, apoptosis and BrdU incorporation of the human P493-6 B cells bearing an inducible MYC construct. Although not unexpected, MPA at clinically relevant levels caused a significant growth inhibition. This inhibition, however, could be partly rescued by guanosine. While guanosine could fully rescue MPA-induced apoptosis, it is unable to rescue the S-phase arrest, indicating a dominant effect of MPA on S-phase arrest.

In summary, we demonstrate that MYC directly regulates genes involved in purine and pyrimidine biosynthesis. The global regulation of virtually all genes involved is consistent with the concept of metabolic control analysis (MCA) which has demonstrated that beyond the traditional concept of rate-limiting biochemical steps, the activation of many points along a biosynthetic pathway is essential for the increased flux of metabolites toward the end product [52]. While increased nucleotide flux is clearly required for normal DNA replication, we surmise that deregulated MYC could create enhanced expression of nucleotide metabolism genes, which would in turn abnormally elevates the nucleotide pools that could contribute to genomic instability. The effects of deregulated nucleotide metabolism pathways downstream of MYC will require additional studies, for which our work has provided a firm foundation.

## Supporting Information

Figure S1 Changes in pyrimidine biosynthetic enzymes gene expression with induction of MYC in HO.15 Myc-ER cells. Time course of gene expression determined by microarray analysis in HO.15 Myc-ER cells with and without 4-OHT. Blue line: without 4-OHT; Red line: with 4-OHT. Solid line: average over replicates, Dashed line = single replicates. Time points are 0, 1, 2, 3, 4, 5, 6, 8,10,12,16, 20 and 24 hours [35].
(0.81 MB TIF)

Figure S2 Changes in purine biosynthetic enzymes gene expression with induction of MYC in HO.15 Myc-ER cells. Time course of gene expression in HO.15 Myc-ER cells with and without 4-OHT as determine by microarray analysis. Blue line: without 4-OHT; Red line: with 4-OHT. Solid line: average over replicates, Dashed line:single replicates. Time points 0, 1, 2, 3, 4, 5, 6, 8,10,12,16, 20 and 24 hours [35].
(1.41 MB TIF)

Figure S3 IMPDH1 and IMPDH2 are directly responsive to MYC induction. (A) Myc binding to target genes in P493-6 cells following MYC induction was measured. The inset shows an immunoblot of Myc expression in P493-6 cells withdrawn from tetracycline. ChIP was performed with P493-6 cells at different time points (0 hr, 6 hr, 10hr, and 26 hr) after withdrawal of tetracycline. Values indicate the percentage of total input DNA. (B) Myc induces IMPDH1 or IMPDH2 expression that correlates direct Myc binding to these genes. Bar graphs represent mRNA expression of each nucleotide synthesis gene relative to 18S rRNA control as determined by real-time PCR in P493-6 cells.
(1.07 MB TIF)

Table S1 Response of nucleotide biosynthetic genes to MYC
(0.06 MB DOC)

Table S2 Primers used for real-time PCR assays
(0.06 MB DOC)

Table S3 Primers used for ChIP assays
(0.04 MB DOC)
