# Supplementary figures and images for: Global Regulation of Nucleotide Biosynthetic Genes by c-Myc
Source: PLoS One. 2008 Jul 16;3(7):e2722. doi: 10.1371/journal.pone.0002722 (PMC2444028; doi:10.1371/journal.pone.0002722)

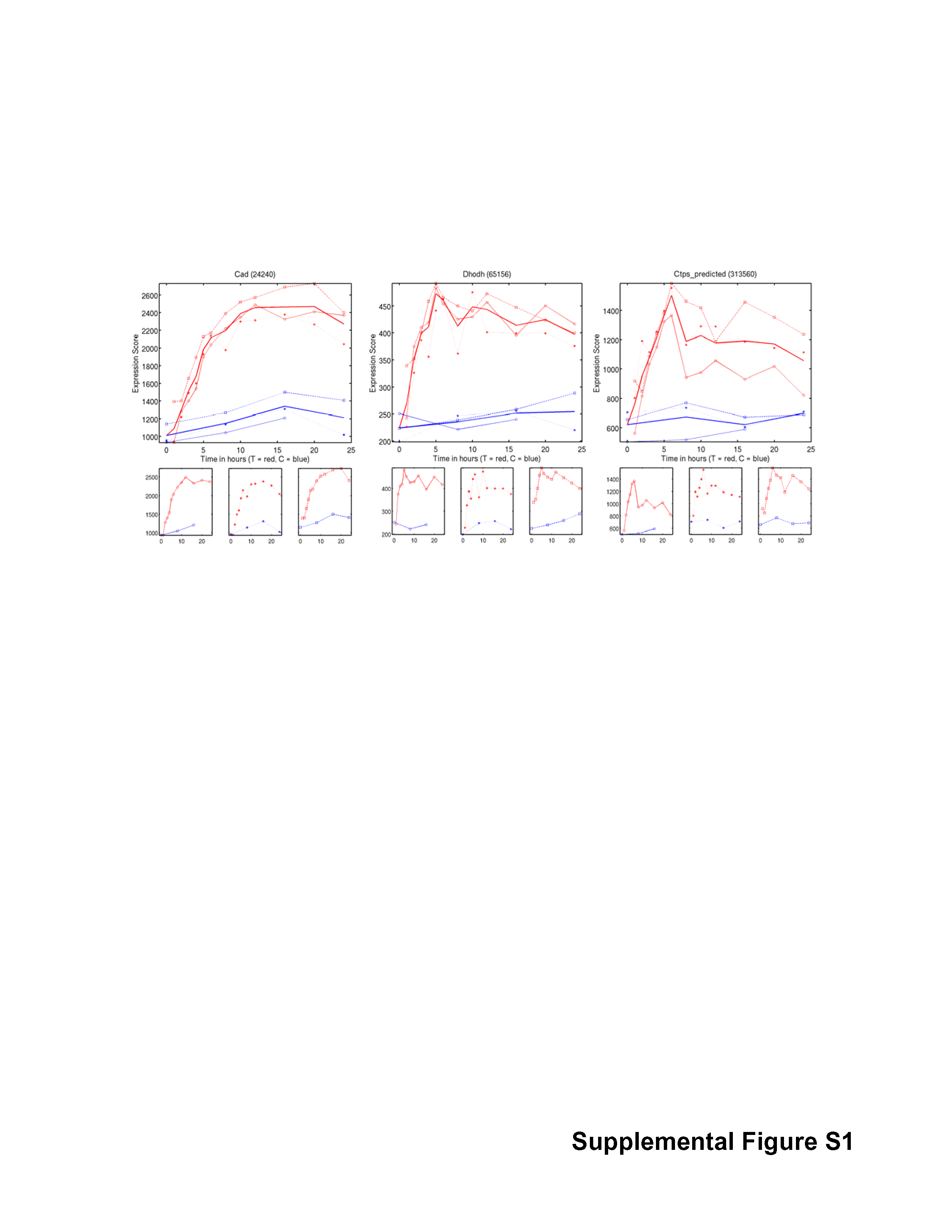

Supplement: Figure S1 — Changes in pyrimidine biosynthetic enzymes gene expression with induction of MYC in HO.15 Myc-ER cells. Time course of gene expression determined by microarray analysis in HO.15 Myc-ER cells with and without 4-OHT. Blue line: without 4-OHT; Red line: with 4-OHT. Solid line: average over replicates, Dashed line = single replicates. Time points are 0, 1, 2, 3, 4, 5, 6, 8,10,12,16, 20 and 24 hours [35]. (0.81 MB TIF) [file pone.0002722.s001.tif]

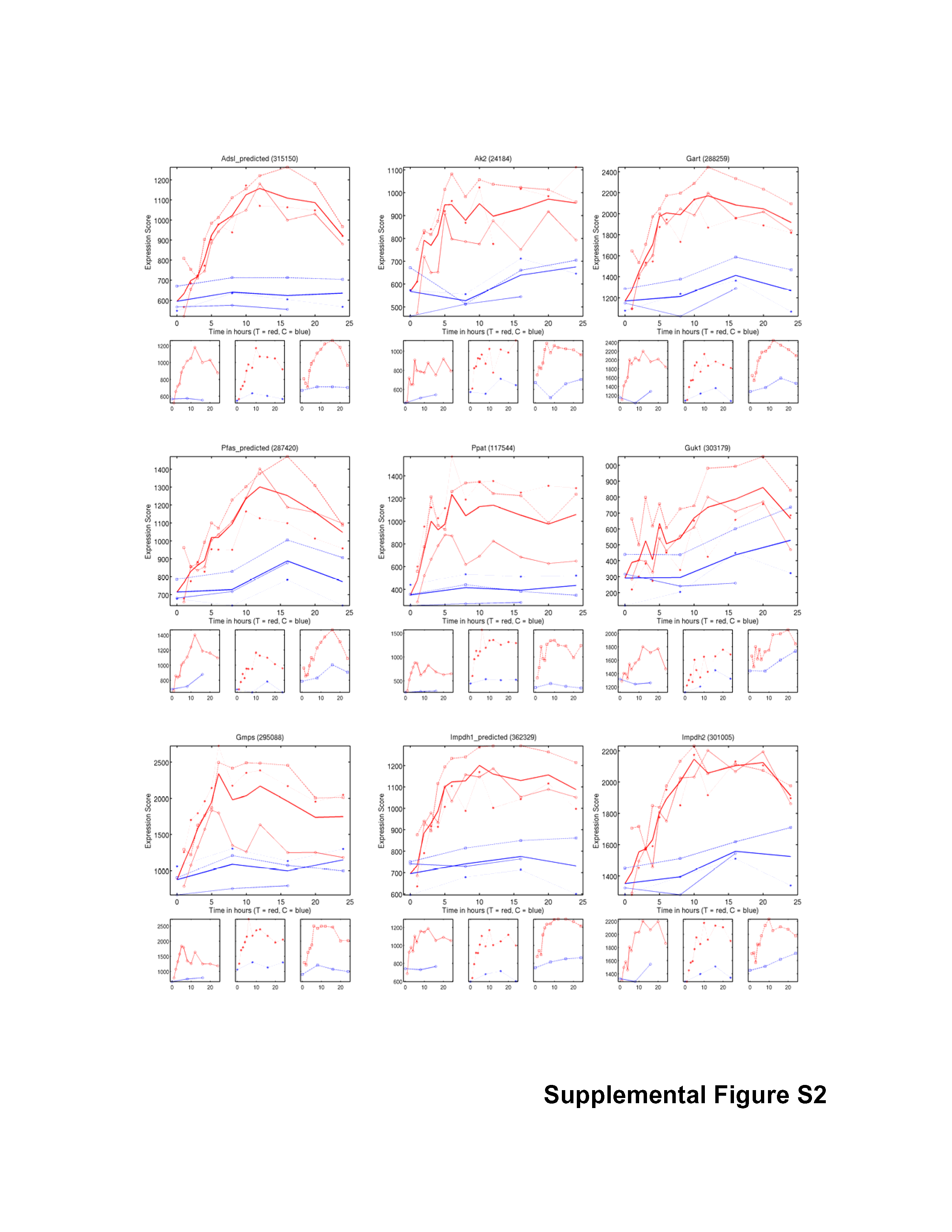

Supplement: Figure S2 — Changes in purine biosynthetic enzymes gene expression with induction of MYC in HO.15 Myc-ER cells. Time course of gene expression in HO.15 Myc-ER cells with and without 4-OHT as determine by microarray analysis. Blue line: without 4-OHT; Red line: with 4-OHT. Solid line: average over replicates, Dashed line:single replicates. Time points 0, 1, 2, 3, 4, 5, 6, 8,10,12,16, 20 and 24 hours [35]. (1.41 MB TIF) [file pone.0002722.s002.tif]

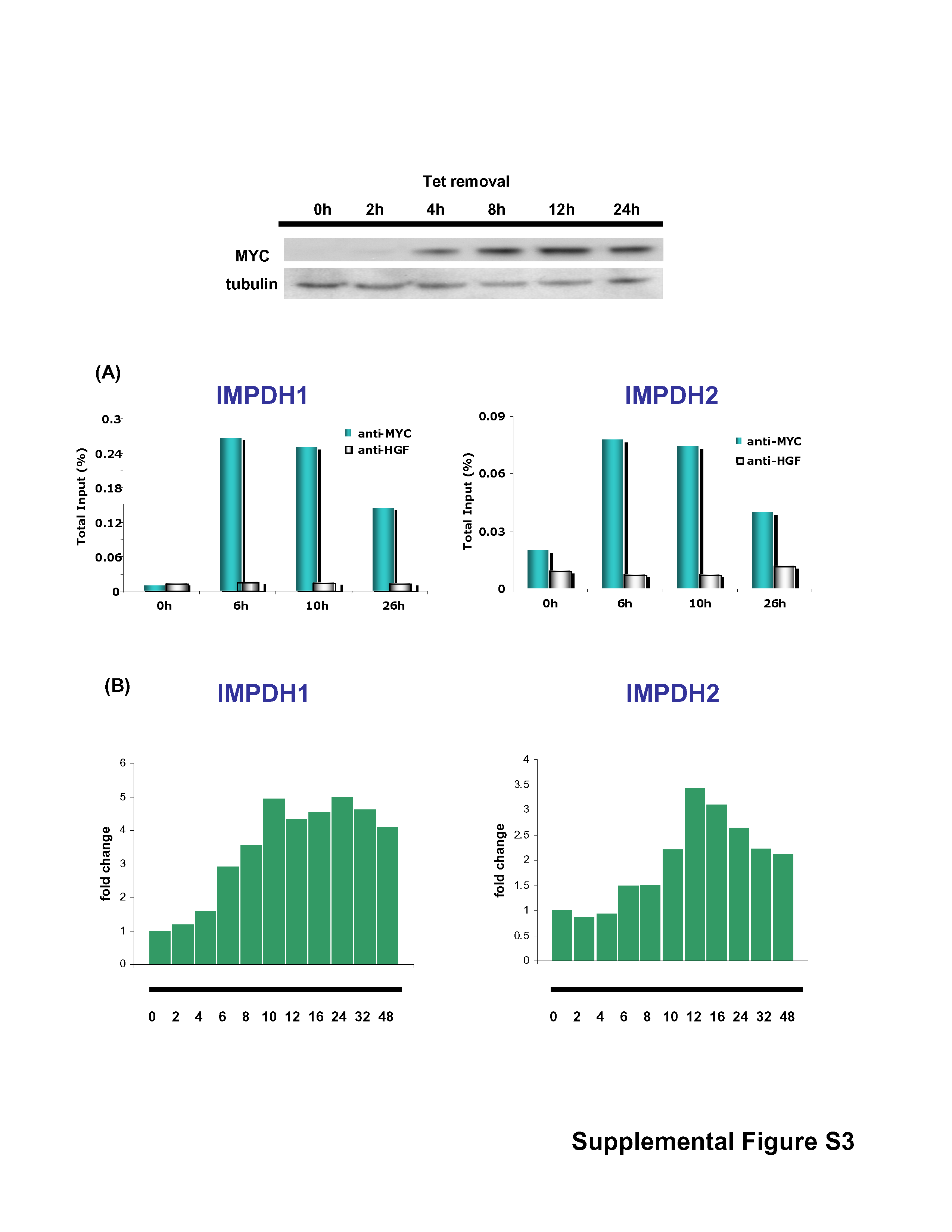

Supplement: Figure S3 — IMPDH1 and IMPDH2 are directly responsive to MYC induction. (A) Myc binding to target genes in P493-6 cells following MYC induction was measured. The inset shows an immunoblot of Myc expression in P493-6 cells withdrawn from tetracycline. ChIP was performed with P493-6 cells at different time points (0 hr, 6 hr, 10hr, and 26 hr) after withdrawal of tetracycline. Values indicate the percentage of total input DNA. (B) Myc induces IMPDH1 or IMPDH2 expression that correlates direct Myc binding to these genes. Bar graphs represent mRNA expression of each nucleotide synthesis gene relative to 18S rRNA control as determined by real-time PCR in P493-6 cells. (1.07 MB TIF) [file pone.0002722.s003.tif]
