# Supplementary material for: Global Regulation of Nucleotide Biosynthetic Genes by c-Myc
Source: PLoS One. 2008 Jul 16;3(7):e2722. doi: 10.1371/journal.pone.0002722 (PMC2444028; doi:10.1371/journal.pone.0002722)
Supplement: Table S1 — Response of nucleotide biosynthetic genes to MYC (0.06 MB DOC) [file pone.0002722.s004.doc]

Table S1. Response of nucleotide biosynthetic genes to MYC

| Gene Symbol | Microarray  Expression  fold change | E box in promoter | E box in intron 1 | ChIP-PET Cluster Size |
| --- | --- | --- | --- | --- |
| ADSL | 3.3-3.7 | 1 | 1 | 2 |
| ADSS | 2.7 | 0 | 2 |  |
| ADSSL1 | NC | 1 | 1 |  |
| AK1 | -3.4 | 1 | 0 |  |
| AK2 | 1.7-3.5 | 0 | 3 | 2 |
| AK3 | 2.7-6.7 | 0 | 1 | 2 |
| AK5 | NC | 0 | 2 |  |
| AK7 | -1.5 | 1 | 0 |  |
| ATIC | 2.9 | 2 | 0 |  |
| CAD | 4.2 | 1 | 2 |  |
| CTPS | 5.2 | 0 | 0 | 2 |
| CTPS2 | NC | 0 | 1 |  |
| DHODH | 1.9-3.0 | 1 | 1 | 2 |
| GART | 2.1-4.0 | 0 | 0 |  |
| GMPS | 4 | 0 | 1 |  |
| GUK1 | NC | 0 | 0 |  |
| IMPDH1 | 2.6 | 1 | 1 | 2 |
| IMPDH2 | 4.9 | 0 | 0 |  |
| NME1 | 7.4 | 0 | 1 | 2 |
| NME2 | 2.2 | 0 | 2 |  |
| NME3 | NC | 0 | 0 |  |
| NME4 | 2 | 0 | 0 | 2 |
| NME6 | NC | 0 | 0 |  |
| NME7 | 1.1 | 0 | 0 |  |
| PAICS | 2.1-10.7 | 1 | 0 | 4 |
| PFAS | 7.7 | 0 | 2 |  |
| PPAT | 6-6.5 | 3 | 0 | 4 |
| RRM1 | 3.1-3.9 | 0 | 0 |  |
| RRM2 | 2.1 | 1 | 0 | 2 |
| UMPS | 2.2 | 0 | 2 |  |
|  |  |  |  |  |

Note: All studies were performed in P493-6 cells. ChIP-PET identification of global MYC binding sites was non-saturating and hence ChIP-PET did not pick up signals for binding that were later identified by quantitative PCR. Microarray expression data are from Zeller et al. (2006) PNAS, ranges resulted from signals with different probe sets NC: No Call
