# Supplementary material for: Global Regulation of Nucleotide Biosynthetic Genes by c-Myc
Source: PLoS One. 2008 Jul 16;3(7):e2722. doi: 10.1371/journal.pone.0002722 (PMC2444028; doi:10.1371/journal.pone.0002722)
Supplement: Table S2 — Primers used for real-time PCR assays (0.06 MB DOC) [file pone.0002722.s005.doc]

**Table S2. Primers used for real-time PCR assays**

| Primers for human cDNA expression | | |
| --- | --- | --- |
|  | FORWARD | REVERSE |
|  |  |  |
| ATIC | GAACCATTGGCGAGGATGAA | GCCTCAGTGAGTAACTCAGGGACT |
| ADSL | GGACCCGCTACAGACAGCAT | CAAACAGATCCGTCGGTTGG |
| CTPS | GATGGCAAGATGCCAATTCTACA | GTTCTGGCATGTCTACGACCAC |
| PFAS | GGATCAAGCCCATCATGTTTAGT | AACCTTTACAACTTCCATGCCTG |
| PAICS | GTCTTCTCTTCGACTACCCAGTGG | CAAATTGAGCTGATCCTTCTGGA |
| PPAT | CAGAGGCAATACCATCTCACCTATAA | ACTCGAATGTGTACCTCTTTTGCAC |
| ADSS | CAAAGTTGGAGTTGCTTACAAGTTAG | CCTGGGAGAGTCTTATATTGAACTTC |
| GMPS | CCAATTAAAACTGTAGGTGTGCAGG | TCTTTACTGGAGATTCCACACACG |
| NME1 | ATGCACTCAGGGCCGGTAG | AGCATGACTCGGCCCGT |
| RRM1 | GTATGCACTTCTACGGCTGGAA | TGGATTGGATTAGCTGCTGGT |
| DHODH | GCAAAGTCAAGCGGGAACTG | TCCAATGGCATCTGTGACTCC |
| IMPDH1 | CCGCAGCCTGTCTGTCCT | CCGACATGGTCCGCTTCT |
| IMPDH2 | AGTGGCTCCATCTGCATTACG | ACCTTGTACACTGCTGTTGCTTG |
| GART | AAGGTTATCCTGGAGACTACACCAA | CAGTCCTAGAGCTTGAGCCTCAG |
| UMPS | TTATTGCGGAAATGAGCTCCA | AGCCATTCTAACCGCTGCTCTA |
|  |  |  |
| Primers for mouse cDNA expression | | |
|  | FORWARD | REVERSE |
|  |  |  |
| IMPDH1 | TGCACGGCCTACACTCTTACG | GCCACCGCTGTCCTCAGTAC |
| IMPDH2 | CATCCAGCATTCCTGTCAGGA | CCCCCGAGTACATCATGGC |
| PPAT* | CCAGAGTCTGCTACGCCTGC | CAGCCCACACTTTGTTGCAT |
| PAICS | TAAGTTCAGGGCCACCCTGA | CTCCACGGCAAGTTGAGTCTC |
| DHODH | CCAGGGTCAAGCGTGAGCT | TCCAATGGCGTCTGTCACTG |
| * Primers for BC023841 | | |

| Primers for rat cDNA expression |
| --- |
| |  | FORWARD | | | | REVERSE | | --- | --- | --- | --- | --- | --- | |  |  | | | |  | | PPAT | AAG AAG AGC TTA TAG CCA ATA AGC CC | | | | CAC AAC GCT GTT TGC TCC AA | | PAICS | AGC CAG CCG AGT GCT TTG |  | |  | GGT GAA CTA GAG GAG CAG ACC G | | DHODH | GAC CAT CCG GGA GAT GTA CG | |  | | CGC TGC TCA CAC CAC CAA C | | IMPDH1 | CCT CAT AGC AGG GAT CCA GC | |  | | TCA TGG ATC GCA GGA CAG AC | | IMPDH2 | GAC GCA GGT GTG GAC GCT |  | |  | GCC AAC ACT TCC TGG GTG AT | | AK2 | ATT GAT GCG TCC CAG ACC C | |  | | TAG GAT GTG GCT TTG GAG AAG G | | GMPS | CTT CAT GAC TGG AGT ACC TGC AA | |  | | CAG TGA CCA TCT TTA GCA CCA CC | | PFAS | TGA TGC TTC GAG GGA TGG A | |  | | AAA TGC CAT GTA ACC TTC CCC | | GART | GCC TAC ACC AAG GGT GTG GA | |  | | GCA TGA AAG ACC TGC AGT CCT | | GUK1 | TGC ACA GGC AGA CAT GGA GA | |  | | CCA GGT TGT CAT TGA CGA TCA C | | CAD | CCT AGT GGG CCA ACA CAT CC | |  | | GTG CGC CAC GTT GAA CAG | | CTPS | CCT GTA GAC GAA GAT GGC TTA GAA C | | | | TCA ATG TCT CCC ACT GTG CC | | ADSL | AAC GTC TCT GAA GGA TTG GTG C | |  | | ATG AAA GGA AGC TCT TGC CG | |
