# Supplementary material for: Global Regulation of Nucleotide Biosynthetic Genes by c-Myc
Source: PLoS One. 2008 Jul 16;3(7):e2722. doi: 10.1371/journal.pone.0002722 (PMC2444028; doi:10.1371/journal.pone.0002722)
Supplement: Table S3 — Primers used for ChIP assays (0.04 MB DOC) [file pone.0002722.s006.doc]

**Table S3. Primers used for ChIP assays**

| ChIP primers for nucleotide synthesis genes | | |
| --- | --- | --- |
|  | FORWARD | REVERSE |
|  |  |  |
| DHODH | TGATGCTCGCGGACTCTTG | GAGATGAGATGCCGCTGCA |
| UMPS | TGCGGGGCATCGTGTCT | CACTCACGGCCCAACTCC |
| CTPS | CGGACGTCGCTCCTACCA | ACAGCCCATTGGGCAGG |
| NME1 | TGCTGGCGGCTGCAG | CCGAACCACCTCTTACCCTTCC |
| PAICS | AGCCCTCAGCCCACTTAGGA | GGGTCAGGGAGACCGTGAA |
| GART | CGGGCATTCCCTTTGGAG | CGGGCCAATTCGGTCTCT |
| PFAS | CCGCGAGTGCATCTTCC | ATGACGAAACGCTGCACC |
| ADSL | TAGCGACAGGTATAAATTCCGGAC | CCGTTACCTGCTCGGCCT |
| ATIC | TGATAAGCCCGGAAACAGCTC | GGATGTGGCGGCTCAGGA |
| ADSS | CGAGGTGGTAATGTACTTCTAACG | AAACTGGAGAGAGTTATCTGAGAGC |
| RRM1 | ACTCTGTCACCTTCTCGATCC | TTCCTTCTCGCCCACC |
| GMPS | AAACCAAGGCACCAGTGG | CGACTAGTAATCGGGAAAGAGC |
| IMPDH1 | CTGTCCCCTTCCTCCTCTCAG | GAAAAGTCTCCCCATAATGCCC |
| IMPDH2 | TTGAACTGGCCGAGGTCCT | GCACATAGTGGCCTTGTCACTG |
|  |  |  |
